# The Castration of Women

**Published:** 1894-03

**Authors:** 


### *EDITORIAL.*

The office of THE JOURNAL is in room 330 Equitable Building

Address all communications, and make all remittances payable to THE ATLANTA MEDICAL AND SURGICAL JOURNAL, Atlanta, Ga.

Articles for publication should reach this office not later than the 15th instant.

The editors are not responsible for views expressed by contributors.

### THE CASTRATION OF WOMEN.

Recent articles and editorials discussing the "Effects of Castration in Women," and "The Great Medical Error of the Day," are very opportune.

In the struggle for supremacy, the rush for honor and desire for fame by devising or originating some new operation, or reporting successful operations by the score, the best interest of the patient is often sacrificed for the benefit of the operator.

While we would do all in our power to encourage and assist the conscientious investigator and operator, we with equal emphasis must condemn him who reports thirty hysterectomies for reflex disturbances, purports to perform bloodless hysterectomy without clamp or ligature, or removes ovaries and tubes for simple reflex nervous phenomena.

We are glad to see so eminent a teacher and author as Dr. Goodell speaking in no uncertain manner of the "Great Medical Error of the Day." There is no doubt that the generative organs of the female have been made the "scapegoat" for ignorance and want of investigation by the *general practitioner* as well as the *specialist.*

The general practitioner failing to recognize diseased conditions often guesses at a diagnosis, while the gynecologist attributes too much importance to local conditions in his eager desire to operate.

But few general practitioners, and fewer gynecologists, understand diseases of the nervous system or recognize their importance, and seek something more tangible on which they can lay the blame for the ailments of women.

Dr. Goodell has certainly graphically pointed out some of the signals by which we may recognize ofttimes the difference between diseased female genitalia and the symptoms produced by "nerve-strain or nerve-exhaustion, coming largely from the frets, the griefs, the jealousies, the worries, the bustles, the carks and cares of life."

"Just as headache does not necessarily mean brain disease, so ovary-ache, groin-ache and backbone-ache do not necessarily mean ovarian disease," etc. It is here the great "Error of the Day" is being committed.

The general practitioner jumps to the conclusion that such symptoms indicate "womb disease," while the gynecologist, examining his patient and finding a deviation from his ideal of the normal, proceeds at once to stretching, sewing up and removing cervix, ovaries or uterus; when, if both classes would take a broader view and inquire more minutely into the history of the patient and trace more accurately from cause to effect there would be less "gynecological tinkering" by the masses, fewer mutilating operations by the gynecologist, and fewer women unsexed for reflex nervous manifestations, for *supposed diseased conditions*.

This naturally leads us to the question:

Does castration abolish the sexual desire of woman?

This question is often asked the gynecologist, to which he cannot and never will be able to give a positive affirmative or negative answer. The stimulus of sexual desire in the female is not located in any one organ, neither is it equally developed in all females. When once the generative organs of the female are fully developed the desire for sexual intercourse may be stimulated by different channels, viz., breasts, clitoris, etc. If removal of the mammary glands or clitoris does not abolish the desire, what might we infer from removal of the ovaries? The ovaries are to furnish the ovule which ripens and discharges without affecting sexual desire in the female, as evidenced by many women bearing children without experiencing such desire. If ovaries were removed in such a case, we would expect the same condition to prevail.

If tubes and ovaries in a previously healthy amorous female be removed near the menopause, we would naturally expect a lessening of the sexual desire, but if in such a case earlier in life the tubes and ovaries became so diseased as to render her an invalid, and are then removed, we could certainly anticipate at least an increase of sexual desire over that existing during her invalid state, especially if she regained her former health and vigor. It is agreed by most writers and operators that removal of the ovaries does not affect the modesty, the figure or the general character of the female; but they are divided as to its effect on sexual desire. Such differences will exist until some definite rule is adopted for classification of cases of castration as regards their age, diseased condition, stage of sexual development or decline. To conclude, we decide:

1st. All things being equal, castration performed at forty would be more likely to produce a decline in sexual desire than if performed at twenty-five.

2d. Castration before complete development is more likely to check sexual desire than if performed after complete development and physiological functions are performed.

3d. Castration for seriously diseased tubes and ovaries, accompanied with severe pain, in a weak, debilitated female, is likely to result in increased sexual desire as she regains her former health and vigor.

